# Embossed Membranes with Vascular Patterns Guide Vascularization in a 3D Tissue Model

**DOI:** 10.3390/polym11050792

**Published:** 2019-05-02

**Authors:** Soyoung Hong, Eun Young Kang, Jaehee Byeon, Sung-ho Jung, Changmo Hwang

**Affiliations:** 1 Biomedical Engineering Research Center, Asan Institute for Life Sciences, Asan Medical Center, Seoul 05505, Korea; skyciel7@gmail.com (S.H.); judy2226@naver.com (E.Y.K.); neonachos@gmail.com (J.B.); 2 Departments of Thoracic and Cardiovascular Surgery, Asan Medical Center, Seoul 05505, Korea; 3 Department of Convergence Medicine, University of Ulsan College of Medicine & Asan Institute for Life Sciences, Asan Medical Center, Seoul 05505, Korea

**Keywords:** embossed membrane, tissue-engineered vascularization, 3D tissue, guided vascularization

## Abstract

The vascularization of three-dimensional (3D) tissue constructs is necessary for transporting nutrients and oxygen to the component cells. In this study, a vacuum forming method was applied to emboss a vascular pattern on an electrospun membrane so that guided vascular structures could develop within the construct. Two- or six-layer constructs of electrospun membranes seeded with endothelial cells and pericytes were stacked and subcutaneously implanted into mice. Blood vessel formation in the implanted constructs with six alternating layers of flat membranes and membranes embossed with a blood vessel pattern was observed after two weeks of implantation. The formation of blood vessels was observed along the embossed blood vessel pattern in the structure of the embossed membrane laminated at four weeks and eight weeks. Vascular endothelial growth factor (VEGF) and angiopoietin 1 (Ang-1) were highly expressed in the vascularized structures. Therefore, we demonstrated that a structure capable of producing a desired blood vessel shape with electrospun membranes embossed with a blood vessel pattern can be manufactured, and that a variety of structures can be manufactured using electrospun membranes in the tissue engineering era.

## 1. Introduction

The fabrication of three-dimensional (3D) thick tissue-engineered structures with vasculature has been an emerging area of tissue engineering and regenerative medicine for decades [1]. However, blood vessels require several weeks to invade small millimeter-sized implants for complete vascularization. Despite this limitation, several techniques for the predetermined vascularization of different cell types in 3D tissue-engineered structures have been developed and studied in tissue engineering and regenerative medicine [2]. Various methods have been used to create channels for vascularization in hydrogels. Muehleder et al. reviewed four types of methods used to create channels in hydrogels: non-sacrificial spacer, sacrificial spacer, photopolymerization-based 3D printing, and planar methods. While their techniques have advantages such as the creation of a defined channel geometry and interconnected channels in hydrogels, the hydrogels are limited by the range of their mechanical properties for making 3D structures.

Electrospinning is a well-known process where high voltages are applied to polymer solutions to generate nanofibers [3]. The polymer fibers are collected in a grounded collector, and an electrical field is applied between a nozzle and the collector, generating a flexible electrospun membrane. The electrospun membrane can mimic the structural properties of the extracellular matrix (ECM), which has a high porosity and surface area [4,5]. In addition, the mechanical properties of the electrospun membrane can be tailored by controlling the geometry of the nanofiber during the electrospinning process. However, because electrospun membranes are highly flexible and thin, they present some challenges for making thick 3D tissue structures. To generate 3D tissue substitutes, we tried to stack up to 11 electrospun membranes; both sides of the polycaprolactone (PCL) electrospun membrane were seeded with endothelial cells and fibroblasts. These were then implanted into a subcutaneous pocket in a mouse [6]. This process resulted in vascularization four weeks after implantation. These results are similar to those observed for polyethylene glycol (PEG)-based hydrogels containing arginyl-glycyl-aspartic acid (RGD) and vascular endothelial growth factor (VEGF) [7].

In order to reduce the vascularization time within the stacks of electrospun membranes, we embossed a predetermined vascularization pattern on an electrospun membrane with a vacuum forming process, stacking with an embossed membrane and a flat membrane, and subsequently generating hollow channels for vascularization. Vacuum forming was used to make patterned sheets with thin membranes of thermoplastic in plastic fabrication [8]. Additionally, we tried to make a vessel pattern of vascularization in the stacked embossed membranes.

To generate a vascular structure with tissue engineering, endothelial cells and perivascular cells are necessary for blood vessel formation [9,10]. The wall of the blood vessel is lined by a thin sheet of endothelial cells, and perivascular cells, such as pericytes and vascular smooth muscle cells, surround the inner endothelium for support and stabilization. In addition, they enhance the culture condition through perfusion or mechanical loading to improve oxygen and nutrient transport throughout the engineered tissue scaffold in the culture of 3D-engineered tissue structures [11,12,13,14].

Here, after fabricating the embossed nanofiber membrane and seeding the layers with endothelial and pericyte cells, the layered embossed membranes were implanted in vivo subcutaneously for perfusion of the 3D tissue structure. We evaluated vascular development along the stem-shaped hollow channels over eight weeks in mice. We hypothesized that the embossed membrane—generated using a vacuum-forming technique after the electrospinning process, followed by alternate stacking of electrospun membranes with an embossed vascular network pattern along with flat electrospun membranes—would induce growth of new vessels into the implant. This would demonstrate the tissue-engineered potential of such structures for generating thick 3D tissues.

## 2. Materials and Methods

### 2.1. Embossed Electrospun Membrane Generation (Vacuum Forming)

An embossed electrospun membrane was created using a vacuum forming method on PCL (PCL, Sigma, MO, USA) electrospun membranes. The PCL membrane was prepared as in a previous study [6]. A 12.5% (*w*/*v*) solution of PCL (80,000 *M*_W_) in 1,1,1,3,3,3-hexafluoro-2-propanol (HFIP, BioPrince, Seoul, Korea) was dissolved with stirring at 80 °C for at least 4 h. The PCL solution was loaded into a 10 mL syringe that was fitted with a 25-gauge blunt needle tip. The solution feed was driven by a syringe pump at a flow rate of 2 mL/h, and an 18 cm working distance and direct current (DC) voltage of 20 kV were applied between the needle tip and the aluminum foil collector using an electrospinning machine (NanoNC Co., Ltd., Seoul, Korea). The electrospun polymer membrane was collected in the form of a random mesh on the collector. The collected PCL membrane was dried overnight in a fume hood. Further drying was achieved within a vacuum chamber to aid the removal of any remaining solvent from the membranes. All membranes were stored in a desiccator until further use. The electrospun membranes were then embossed with vacuum forming. A master mold with patterns of SU-8 250 µm height was prepared by MicroFIT (Seongnam-si, Gyeonggi-do, Korea). A polydimethylsiloxane (PDMS) (Dow Corning, Seoul, Korea) pre-polymer mixture was prepared at a 10:1 base to curing agent ratio. The PDMS mold for an embossed membrane was cast against the master mold and thermally cured at 60 °C for 2 h to obtain a positive replica mold with embossed channels 250 µm in height.

**Figure 1:**
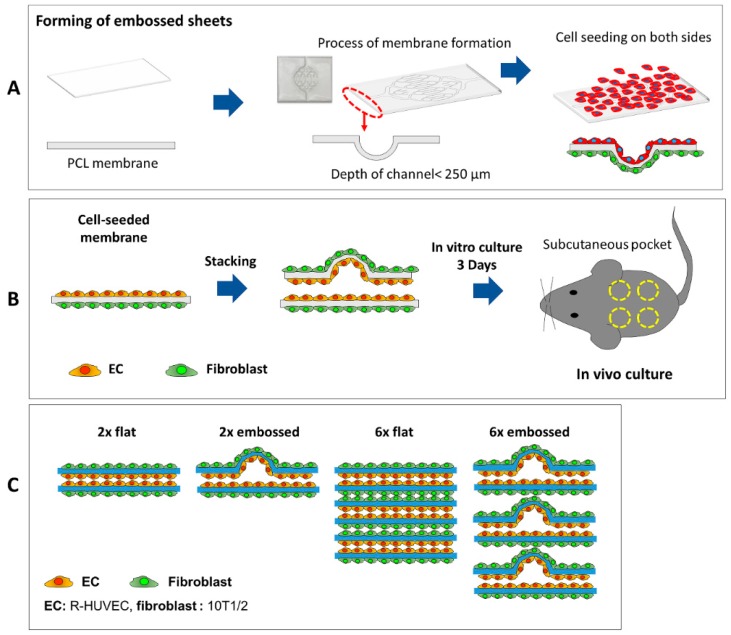
Generation of electrospun membranes with embossed patterns for guided vascularization of seeded cells. (**A**) Schematic diagram of forming of embossed sheet followed by seeding with human endothelial and mouse fibroblast cells. (**B**) Cell-seeded embossed sheets were stacked and cultured for 3 days before implantation into subcutaneous pockets in mice. (**C**) Experimental groups for in vivo experiment included stacks of two flat membranes (group 2× flat), an embossed membrane stacked on a flat membrane (group 2× embossed), six layers of flat membranes (group 6× flat), and six layers of alternating flat and embossed membranes (group 6× embossed).

Vacuum forming was carried out on a homemade setup with the PDMS mold, as shown in Figure 1 and Figure S1. Briefly, a food storage container was used for a vacuum forming chamber, and a hole was drilled in one side of the container. A connector of vacuum tubing was bonded to the hole in the container. The PDMS pattern mold was mounted in the storage container, and the electrospun membrane was laid directly onto the PDMS mold. The top of the PCL membrane was sealed with plastic wrap to make an enclosed chamber. A vacuum (−0.56 bar) was applied to assist in forming the membrane at 55 °C for 1 h. The vacuum forming was carried out with a laboratory vacuum network. The embossed electrospun membranes were observed by SEM (AIS2000C, Seron Technologies, Uiwang-si, Gyeonggi-do, Korea).

### 2.2. Preparation of Cell Sheet for Cell Culture

After removal from the vacuum forming chamber, membranes were bonded to a frame for layering, which was used in a previous study [6,15]. Frames were fabricated with a 10 × 10 mm^2^ square window with a polyethylene terephthalate (PET) sheet by Sahm-Oh Precision Machining (Seoul, Korea). These frames were washed with 70% (*v*/*v*) ethanol (EtOH, Duksan, Ansan-si, Korea) three times and then air dried. The embossed and the flat membranes were bonded onto the sterilized frames with a medical silicone bond (Dow Corning) and dried in a fume hood (the total number of each type of membrane, *n* = 394) (Figure S2A). For sterilization, the membranes with frames were washed with 70% ethanol three times, and then each side of the membrane was exposed to UV light for 30 min and then washed with phosphate-buffered saline (PBS, pH 7.4, Thermo Fisher Scientific, Waltham, MA, USA) once. All membranes were coated with human fibronectin (Corning) in PBS at 1 µg/cm^2^ at room temperature (RT) for 1 h and then stored in a sterile container at 4 °C until use. The fibronectin-coated membranes were immersed in culture medium before cell seeding.

### 2.3. Cell Culture

**Figure 2:**
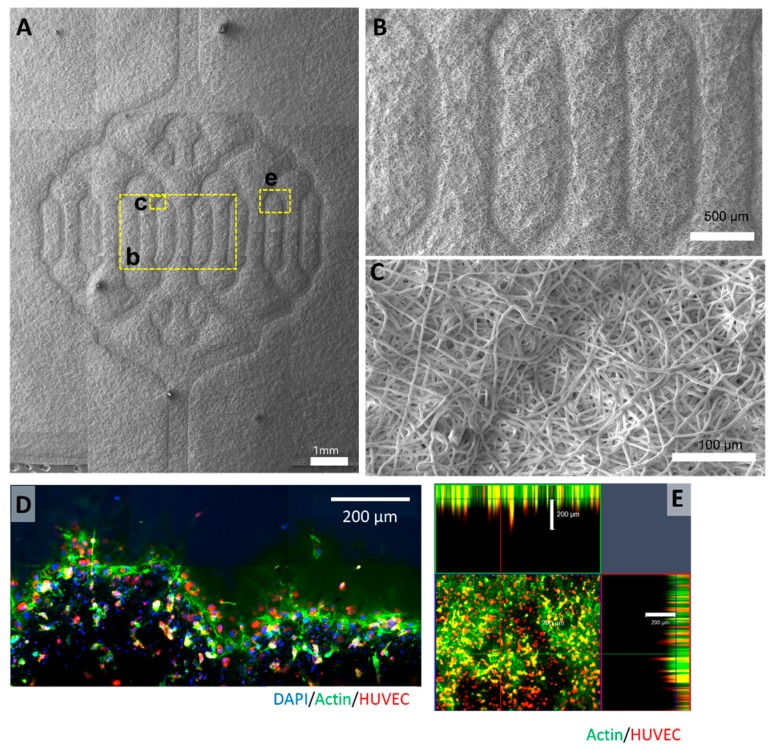
Embossed polycaprolactone (PCL) membrane. (**A**) SEM imaging shows embossed pattern of vacuum-formed electrospun PCL membrane. (**B**,**C**) Further magnification of formed PCL embossed membrane shows detail of embossing and woven electrospun PCL fibers. (**D**) Confocal image and (**E**) orthogonal views of human umbilical vein endothelial cells (HUVECs) on embossed PCL membrane on day three. Scale bars are 200 μm. Green: F-Actin, red: HUVEC, blue: DAPI.

Red fluorescent protein (RFP)-expressing human umbilical vein endothelial cells (R-HUVECs) (Olaf Pharmaceuticals, Worcester, MA, USA) and mouse embryo fibroblast cells (10T1/2) were used in this study. R-HUVEC cells were cultured in endothelial cell growth medium (EGM-2, Lonza, MD, USA) supplemented with 1% (*v*/*v*) Antibiotic-Antimycotic solution (Thermo Fisher Scientific, Waltham, MA, USA). Mouse embryo fibroblast cells (10T1/2) were cultured in Dulbecco’s Modified Eagle Medium (DMEM, Thermo Fisher Scientific) supplemented with 10% (*v*/*v*) fetal bovine serum (FBS, Thermo Fisher Scientific) and 1% (*v*/*v*) Antibiotic-Antimycotic solution at 37 °C in a 5% CO_2_ humidified atmosphere. Both cell types were harvested using 0.25% (*w*/*v*) trypsin/Ethylenediaminetetraacetic acid (EDTA) (Thermo Fisher Scientific), resuspended in media at a density of 1–2 × 10^6^ cells/500 µL over the electrospun membrane, and incubated at 37 °C in a 5% CO_2_ humidified atmosphere for 2 h to permit cell adhesion. Pericytes were then seeded at the same density of 1–2 × 10^6^ cells/500 µL on the opposite side of the membranes using the same procedure as described above. Endothelial cells were seeded onto the negative pattern surface, whereas pericytes were seeded onto the positive pattern surface. All cell-seeded membranes were placed in ultra-low adherent 6-well culture plates (SPL, 32006, Pocheon-si, Korea) with 2 mL of medium per well and incubated at 37 °C and 5% CO_2_ (Figure S2B,C). The next day, a frame rig was used for fast and multilayer stacking of the membranes (Figure 2D). As shown in Figure 1C, a flat membrane and an embossed membrane or two flat membranes were laminated based on the endothelial cell-seeded surfaces to make a pair. The 2- or 6-membrane structures were assembled for stacking (Figure S2D). In all cell-cultured membranes, a stitch was made with surgical thread (3-0 Mersilk, Ethicon, Somerville, NJ, USA) at every fourth corner to gather the layered membranes. Table 1 provides a description of the experiments.

### 2.4. In Vivo Experiments

The animal experimental protocol was reviewed and approved by the Institutional Animal Care and Use Committee of Asan Medical Center (protocol number: 2016-02-234). All institutional and national guidelines for the care and use of laboratory animals were followed. Eight-week-old male nude mice were housed in an animal room in a specific pathogen-free facility with controlled temperature and relative humidity.

For implantation, mice were anesthetized with isoflurane, and two subcutaneous pockets were made to both the left and the right of the midline of the backs of the mice via 1.5 cm incisions (Figure 1B, *n* = 16 mice). The multilayered membranes were implanted in the subcutaneous pockets. The implanted tissue sheets were harvested with the surrounding tissue 1, 2, 4, or 8 weeks after implantation and then fixed in 4% paraformaldehyde at 4 °C overnight and embedded in optimal cutting temperature (OCT) compound (Sakura Finetek USA INC., Torrance, CA, USA), frozen, and stored at –80 °C. We subsequently used a cryostat to obtain 10 µm sections, which were stained using standard hematoxylin and eosin (H&E) staining methods. Sections were viewed using routine bright-field light microscopy.

### 2.5. Harvesting and Fixation of Implanted Tissue Sheets

To identify the direct connections between host vessels and the embossed vascular pattern within the transplant, fluorescein-conjugated dextran (Sigma, 70 kDa; 0.5 mL of 100 mg in saline) was intravenously injected into the tail veins of mice and circulated for 2 min [15,16]. The mice were euthanized with CO_2_ inhalation, and the tissue sheets were harvested and fixed by incubation in 4% paraformaldehyde (*n* = 4, every time point) (PFA, Biosesang, Seongnam-si, Korea) for 24 h and then kept in 30% sucrose at 4 °C until the samples sank. The harvested sheets were transferred to an embedding mold containing OCT cryostat embedding compound (Tissue-Tek, Torrance, CA, USA) and then frozen in the cryostat chamber. The frozen blocks could then be stored at −80 °C for months. The implanted structures from the mice with non-perfused of Fluorescein isothiocyanate (FITC)-dextran were cut into 10 μm-thick sections and dried on a 26 °C hot plate (*n* = 12–15). The sections could then be stored at −20 °C for months.

### 2.6. Immunofluorescence Staining

The three sections for each immunofluorescence staining were used after the observation of sectioned images under light microscopy. The sections were blocked and incubated for 1 h at 37 °C with the following primary antibodies: anti-VE-cadherin, anti-CD31, anti-angiopoietin 1 (anti-Ang-1), anti-VEGF, and anti-human mitochondria (Table 2). We selected and stained the sections of the marked region (Figure S3a). The sections were then further incubated for 1 h at RT with either Alexa Fluor 488 goat anti-mouse or goat anti-rabbit secondary antibodies (1:100). The F-actin of the tissue sheets was visualized with a 1:40 dilution of Alexa Fluor 488 phalloidin, and cell nuclei were counterstained using 4’,6-diamidino-2-phenylindole (DAPI) nucleic acid stain. Sections were viewed using an LSM710 confocal microscope, and images were captured and analyzed with digital software (Zen2011, Carl Zeiss) and FIJI/ImageJ (National Institutes of Health, Bethesda, MD, USA) software.

### 2.7. Quantification of Immunofluorescence Staining

Fluorescence quantification was performed using FIJI/ImageJ2 software (Supplemental data) [17]. We defined regions of interest (ROIs, transplanted embossed tissue region) in three immunofluorescence figures for every group and obtained the area fraction for each ROI and each molecule (Ang-1) [18,19,20] to indicate their expression levels per frame analyzed. We also defined ROIs for each fluorescent area and divided them by the DAPI ROI area to obtain the percent area data and the area fraction [6,20].

### 2.8. Statistical Analysis

All data are reported as the mean ± SD. Experimental groups were compared with a one-way ANOVA of origin and two-sample *t*-tests using Microsoft Excel 365 (Redmond, WA, USA), and *p* < 0.05 was considered statistically significant.

## 3. Results

### 3.1. Forming of Embossed PCL Electrospun Membrane and Cell Culture on Embossed Membrane

The embossed membrane was observed with SEM (Figure 2). The embossed pattern was observed on the surface of the PCL membrane clearly, and the magnification of Figure 2A is shown in Figure 2B,C. The PCL microfiber of the embossed membrane was distributed randomly and pressed along the pattern of the PDMS mold (Figure S1B,C).

After the embossed membranes were prepared using the optimized PCL concentration and the vacuum forming temperatures, the endothelial cells were placed on the embossed surfaces. Confocal microscopy images revealed that R-HUVECs were aligned and distributed onto the embossed electrospun membrane at day three after seeding (Figure 2D,E). Confocal images of the embossed membrane showed that the embossed vascular patterns were 208.8 ± 44.5 µm in height, which means that the embossed pattern was maintained three days after culture.

### 3.2. Gross Observations

**Figure 3:**
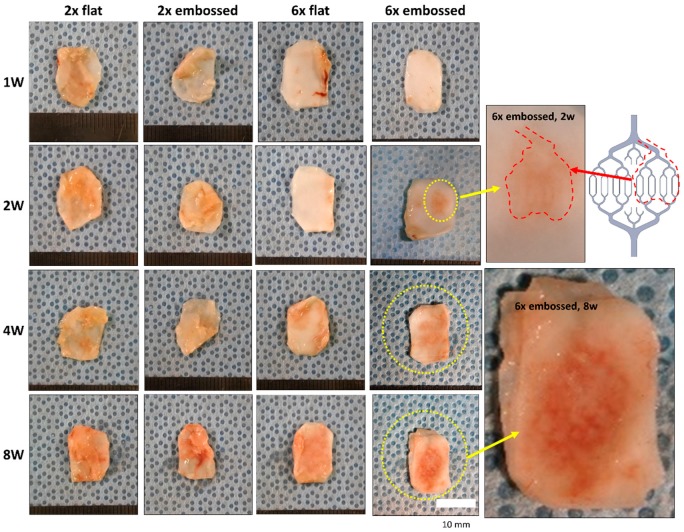
Embossed sheets after subcutaneous implantation in mice at one, two, four, and eight weeks.

All experimental sheets were confirmed to be intact at the surgical sites at postoperative weeks one, two, four, and eight. Representative photographs (Figure 3) indicated that all layered PCL membranes were covered with host-driven connective tissue one week after surgery. Specifically, in the two-membrane group (groups 2× flat and 2× embossed), connective tissue was observed to be thicker than in the six-layered membrane constructs (groups 6× flat and 6× embossed) at one and two weeks.

The preformed vascular patterns were observed on the outer surface of the stacked PCL membranes at week two in group 6× embossed. At four weeks and eight weeks, a clear vascular pattern appeared outside the construct in group 6× embossed.

### 3.3. Analysis of Vascularization

**Figure 4:**
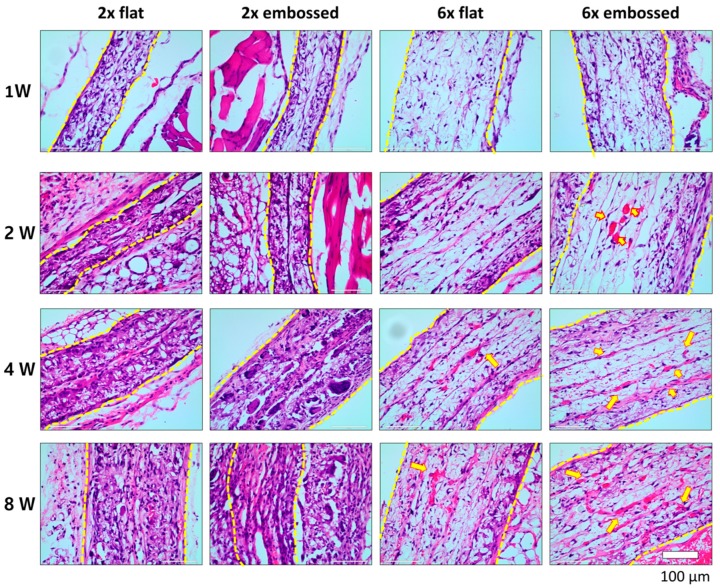
Hematoxylin and eosin-(H&E) stained cross-section images of experimental groups at one, two, four, and eight weeks after implantation. Arrowheads show red blood cells indicating blood vessel formation. Dashed lines indicate edge of transplanted embossed sheets.

The results of histological imaging were consistent with the gross morphological analysis (Figure 4). In H&E images of group 6× embossed, red blood cells were found inside the construct at two weeks, indicating blood vessel formation. In Figure S3, red blood cells were stained in the middle part of group 6× embossed at two weeks, and it was confirmed that the red blood cells were recruited in along the pre-guided pattern two weeks after implantation. At four weeks, blood vessel formation was also observed in group 6× flat. In images of groups 2× flat and 2× embossed, which contained two-layered membrane implants, red blood cells were not found over the implantation period. In groups of two-layered membrane implants, a fibrotic capsule formed around the implant, and at two, four, and eight weeks, it was difficult to differentiate between the implanted PCL membrane and the surrounding tissue. The cells were infiltrated into the implanted PCL membranes. However, in groups 6× flat and 6× embossed, which comprised six membrane layers, the PCL membrane was not degraded, and it remained tight until eight weeks.

**Figure 5:**
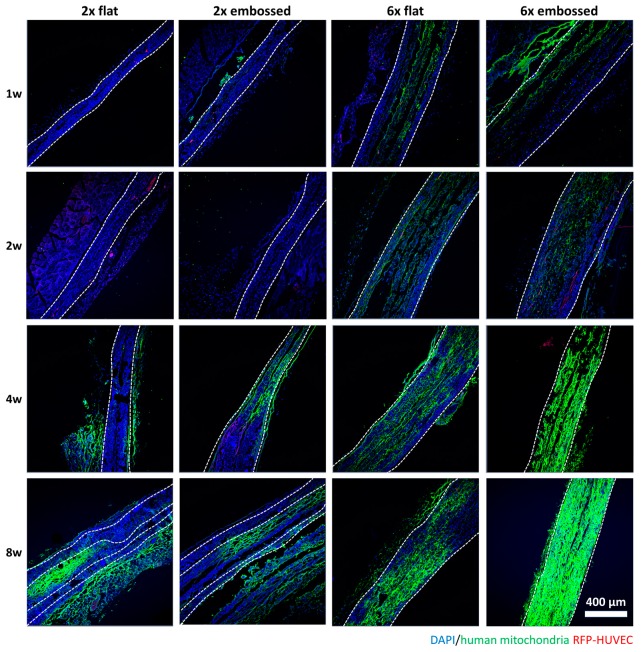
Immunofluorescence images showing differences in human mitochondria expression in cross-sections of experimental groups at one, two, four, and eight weeks. Dashed lines indicate edge of transplanted embossed sheets. Green: human mitochondria, red: HUVEC, blue: DAPI.

In Figure 5, human mitochondria were not expressed in overall images of either 2× flat or 2× embossed groups until two weeks, but they were expressed in peripheral tissues after four weeks. Conversely, in groups 6× flat and 6× embossed, mitochondria were observed in the middle of the constructs from week one, and after four weeks, mitochondrial expression was seen throughout the entire construct in the group 6× embossed implants

VE-cadherin (Figure S4), CD31 (Figure S5), and human mitochondria (Figure 5) were stained to confirm the characteristics of the cells inside the implanted construct. In Figure S4, VE-cadherin expression could be seen along the edges of the PCL membranes at one and two weeks after implantation in the six-layered membrane implants, while two-layered membranes (group 2× flat and 2× embossed) did not show VE-cadherin expression in the middle area of the implanted membranes. In groups 2× flat and 2× embossed, the expression of VE-cadherin was observed after four weeks. At eight weeks, the expression of R-HUVEC in peripheral tissues could be confirmed in two-layered membranes. In all groups, almost no CD31 expression was observed until after two weeks, but expression was confirmed after four weeks (Figure S5). The used anti-CD31 antibody has been said to have reactivity with human cells, which means that in the early periods of the in vivo experiment, cells were recruited from the implanted host, and then implanted human cells subsequently participated in vascularization in the last part of the experiment [21].

### 3.4. Vascular Maturation and Anastomosis of Vascularization

**Figure 6:**
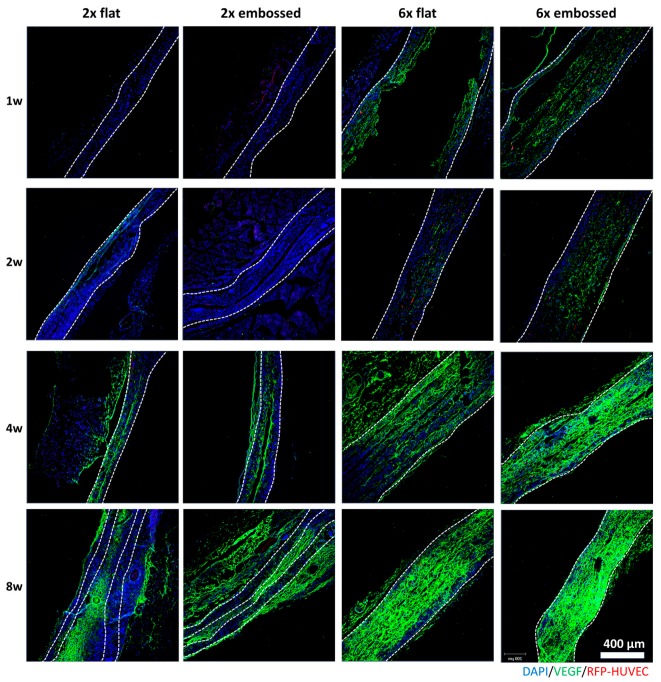
Immunofluorescence images showing differences in VEGF expression in cross-sections of experimental groups at one, two, four, and eight weeks. Dashed lines indicate edge of transplanted embossed sheets. Green: VEGF, red: HUVEC, blue: DAPI. Scale bars are 200 μm.

**Figure 7:**
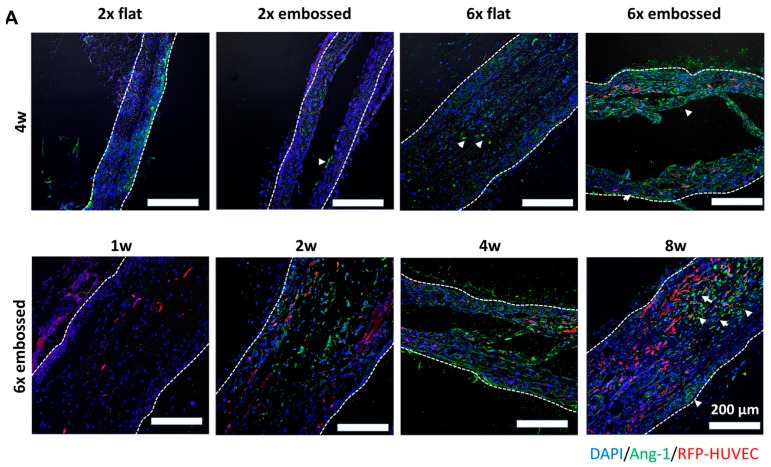

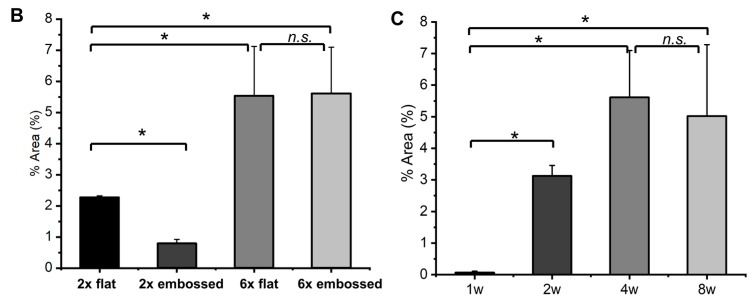
(**A**) Immunofluorescence images showing differences in Ang-1 expression in cross-sections of experimental groups at one, two, four, and eight weeks. Dashed lines indicate edge of transplanted embossed sheets. Arrowheads indicate Ang-1 expression in images after staining. Green: Ang-1, red: HUVEC, blue: DAPI. Scale bars are 200 μm. (**B**) Mean percent area of Ang-1 at four weeks within implanted layered sheet regions of interests (ROIs) in each experimental group. (**C**) Mean percent area of Ang-1 expression of group 6x embossed at one, two, four, and eight weeks. (*n* = 4; data represent average ± SD, * *p* < 0.05).

VEGF and angiopoietin 1 (Ang-1) promote microvascular formation and vessel formation in vascular development [22,23]. To confirm vessel formation and maturation, immunostaining of VEGF (Figure 6) and Ang-1 (Figure 7A and Figure S6) was conducted with implanted embossed sheets. In both six-layered membrane groups, but not the two-layered membrane groups, VEGF expression was observed at one week. VEGF expression of group 6× embossed was broadly distributed compared to that of group 6× flat. Ang-1 is one of the promoting proteins in the vascular maturation process [24,25]. The expression of Ang-1 was observed with confocal images after immunostaining (Figure 7A) and analyzed quantitatively (Figure 7B,C), especially when the level of Ang-1 expression in group 6× embossed was increased from two weeks, and it was confirmed that there was no significant difference between levels at four and eight weeks post-implantation.

**Figure 8:**
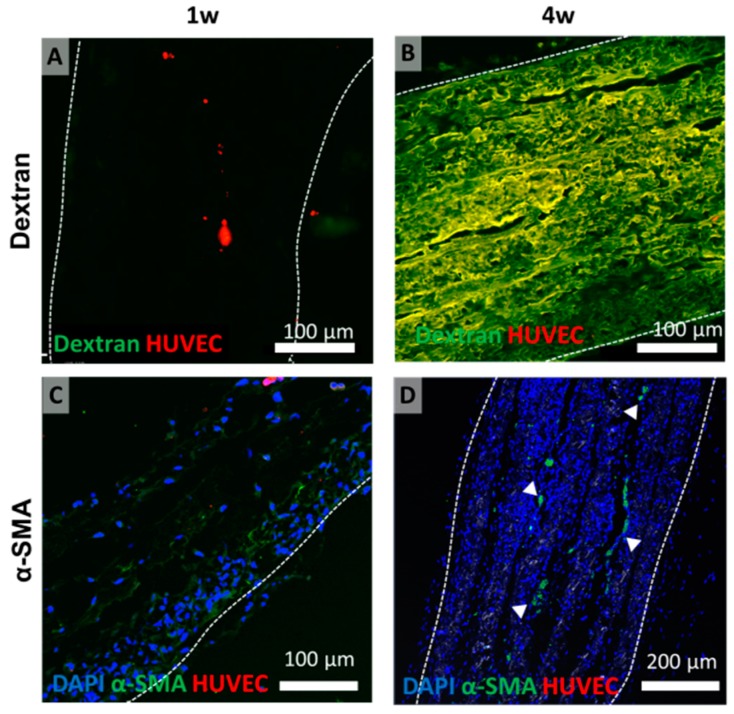
(**A**,**B**) Fluorescent images showing differences in Fluorescein isothiocyanate (FITC)-dextran perfusion, indicating connection between host and implanted sheets (group 6× embossed) in vivo. Dashed lines indicate edge of transplanted embossed sheets. Green: FITC-dextran, red: HUVEC. (**C**,**D**) Immunofluorescence images showing α-SMA expression in cross-sections of group 6× embossed at one and four weeks. Green: α-SMA, red: HUVEC, blue: DAPI. Arrowheads indicate α-SMA expression.

Among all the experimental groups, the expression of FITC in the implanted structure was observed in group 6× embossed, but fluorescence was not observed at one week (Figure 8A,B). This may have been due to the connection of host vessels to the embossed channels within the implanted structure. However, in groups of two-layered membranes, the FITC could be seen in the fibrous capsule of the construct, but it was difficult to determine whether it was expressed clearly inside. This confirmed that some of the inserted structures of group 6× embossed were directly connected to the host, while the blood vessels were not directly connected in the 2× flat, 2× embossed, and 6× flat groups. The expression of α-SMA was observed in the process of blood vessel maturation [26]. At one week, α-SMA was not expressed clearly in any implanted tissue sheets. The expression of α-SMA was confirmed in the middle region of only one 6× embossed sample at four weeks but not in the other groups by immunostaining (Figure 8C,D, and Figure S7).

## 4. Discussion

In this study, we utilized an electrospun membrane with an embossed pattern to promote vascularization by a method that has not been reported elsewhere. Previous studies generated embossed membranes using an electrospinning process performed in a patterned collector composed of metal protrusions or a wire net to obtain a membrane protruding only from the pattern portion [27,28,29]. However, the electrospun membrane on the metal patterned surface could be generated with a protruding pattern shape in which electrospun fibers combined with aligned nanofibers along the metal pattern, making it difficult to obtain an embossing surface with electrospun fibers. Vacuum thermo forming—where a thermo-polymer is heated to a forming temperature, stretched onto a mold, and forced against the mold by a vacuum—was used to produce the embossed electrospun membranes. Various temperatures and concentrations of the polymer material were used to identify the conditions that would make the membranes take the shape of the mold. In addition to this, if the solvent used or the spinning conditions were changed, the shape of the membrane sometimes appeared in various forms (Figure S8) because the mentioned conditions affected the surface topography of the electrospun membrane [30].

In our experiment, since the vacuum forming process was conducted after the generation of the electrospun membrane, the surface topography of the electrospun membrane was a key factor affecting the creation of the embossed membrane. As for the conditions of the vacuum forming process, 12.5% (*w*/*v*) PCL solution in HFIP solvent was electrospun and then formed into the embossed membrane at 55 °C for 1 h.

In this study, the thermoformed membrane pattern was intended to provide a scaffold for the vascularization of cells attached to the membrane. Adhesion of vascular endothelial cells was observed, confirming that endothelial cells were well adhered to the embossed surface (Figure 2). After three days of static culture, perfusion culture was performed in vivo.

In the animal experiment, we used subcutaneous pockets of mice as in vivo bioreactors. Similarly, there have been studies in which the constructs were immediately implanted into animals, and results were observed [31,32,33]. The implantation in animals has the advantage that it allows the evaluation of large volumes, complex structures, and high regenerative capacities [34].

In animal studies, anastomosis between host vessels and implanted extracellular (EC) network structures occurs as early as two weeks post-implantation [35,36]. Here, similar results were achieved when HUVEC and 10T1/2 mesenchymal precursor cells were seeded on the embossed membrane structure, and red blood cells were confirmed within the structure by H&E staining. The H&E-stained images showed that red blood cells were present along the embossed blood vessel pattern (Figure S3). In groups consisting of a single pair of membranes, a fibrotic capsule formed before connections with host blood vessels were formed, and blood vessels inside the implanted tissues were not generated. We confirmed that connected blood vessels formed along the hollow connected channels of the membrane at between one and two weeks post-implantation. When the implanted structure was surrounded by fibrotic tissue, we observed no anastomosis within the implants.

Increased human mitochondria expression was confirmed by immunofluorescence staining in all groups after four weeks. The transplanted human endothelial cells gradually proliferated and played a role in the generation of vascular structures within the implants. Human mitochondria and human CD31 were predominantly seen in implanted structures four weeks after implantation, compared to human mitochondria and CD31 expression of one and two weeks. Additionally, VEGF expression was increased four weeks after implantation, which was related to the increase in human CD31 and mitochondria expression. These data are related to the fact that the percent area of Ang-1 was increased at four weeks compared to one week. Ang-1 is one of the promoting proteins in the vascular maturation process, and it is required for the correct organization of newly formed vessels [24,25]. In particular, vascular remodeling and maturation are known to activate the tie2 receptor of Ang-1 during vascular maturation [37]. These findings suggest that the implanted human endothelial cells did not contribute directly to the early stages of vascularization; rather, host mouse cells or implanted mouse 10T1/2 cells might have been actively recruited and proliferated in the implanted structure and promoted the vascularization at the early stages.

Additionally, α-SMA was localized in the center position of the implanted tissues at four weeks, while α-SMA expression at one week was not observed. The expression of α-SMA was observed in the process of blood vessel maturation [26]. These results suggest that the maturation process of vascularization may be induced only four weeks after implantation. However, we could not detect α-SMA expression at eight weeks (Figure S7). Considering that α-SMA expression was confirmed in only one group 6× embossed sample at four weeks, it can be inferred that vessel maturation does not occur in the whole blood vessel formed in the implanted membrane but only in a specific part.

In this study, we tried stacking multi-embossed membranes with human EC and mouse 10T1/2 mesenchymal precursor cells. The 10T1/2 cells were reported to have the ability to differentiate into mural cells through heterotypic interaction with endothelial cells [38,39,40]. In addition, Hirschi et al. reported that 10T1/2 cells have the capacity to become incorporated into developing vessels in vivo in response to endothelial cells [41]. The PCL embossed membrane showed vascularized blood vessels in the middle of the stacked area after subcutaneous implantation. The use of embossed space on the membrane can guarantee a defined channel structure to yield adequate conditions of mass transport throughout a laminated membrane. This technique has also been used in the design of biocompatible hydrogels with removable spacers in vitro [42,43,44,45,46]. Here, we studied the embossed membranes in vivo for up to eight weeks. Additionally, implanted membranes remained intact at the end of the animal experiment. Thus, embossed membranes will be useful for vascularized tissue engineering, as they can maintain their structure and mechanical properties during vascularization.

## 5. Conclusions

Creation of engineered tissue through guided vascularization is a key challenge in tissue engineering and regenerative medicine. To obtain a functional vasculature consisting of adequate vessel dimensions, embossing of a vascular pattern through a vacuum forming process after generation on PCL electrospun membranes was created. The embossed membranes with vascular pattern exhibited accelerated vascularization in cell-seeded multilayered membrane structures. In addition, endothelial cells and supporting cells interacted with vessel generation and maturation in the guiding vascular patterns in vivo. Bringing together vascular-shaped channels and multiple cell types, including endothelial cells, may enable the vascularization of engineered tissues in vivo.

## 6. Patents

Korea patent: 10-1829132, three-dimensional tissue regeneration with preformed thin membranes.

## Figures and Tables

**Table 1:** Experimental groups.

Group Name	Number of Embossed Membranes in Laminated Assembly	Number of Assembled Membranes	Number of Stacked Structures
2× flat	0	2	16
2× embossed	1	2	16
6× flat	0	6	16
6× embossed	3	6	16

**Table 2:** Materials used for immunofluorescence staining.

Name	Type	Dilution	Company, No.	Annotation
Anti-VE-cadherin	poly	1:100	Abcam Ab33168	Endothelial cell marker
Anti-CD31	mono	1:100	Thermo MA3100	Endothelial cell marker
Anti-Ang-1	poly	1:100	Abcam Ab8451	Vascularization
Anti-VEGF	mono	1:100	Abcam Ab1316	Vascularization
Anti-Human mitochondria	mono	1:100	Sigma MAB1273	Human cell marker
Anti-α smooth muscle actin	poly	1:100	Abcam Ab5694	Vessel maturation
Phalloidin	-	1:40	Thermo R415	F-actin
4’,6-Diamidino-2-Phenylindole, Dihydrochloride		1:100	Thermo D1306	Cell nuclear staining
Alexa Fluor 488 goat anti-Mouse	poly	1:100	Thermo A11001	2nd antibody
Alexa Fluor 488 goat anti-Rabbit	poly	1:100	A11008	2nd antibody

* VEGF = vascular endothelial growth factor, Ang-1 = angiopoietin 1.

## Supplementary Materials

The following are available online at https://www.mdpi.com/2073-4360/11/5/792/s1, Figure S1: (A) Schematic of vacuum forming process for generation of embossed membrane. (B) Embossed membrane attached on polydimethylsiloxane mold. (C) Generated embossed polycaprolactone membranes. Supplemental data: Quantification of immunofluorescence staining using ImageJ software (% Area); Figure S2: Preparation of cell sheet for cell culture. (A) Embossed membrane bonded onto frame having two notches, and (B) prepared in 6-well culture plate. (C) Cells were seeded onto both sides of membrane according to experimental procedures. (D) Stacking of embossed membrane was conducted using frame rig for easy assembly; Figure S3: Hematoxylin and eosin-stained cross-section images of group 6x embossed after 2 weeks of implantation. (A) Region used for observation; Figure S4: Immunofluorescence images showing differences in VE-cadherin expression in cross-sections of experimental groups at 1, 2, 4, and 8 weeks. Dashed lines indicate edge of transplanted embossed sheets. Green: VE-cadherin, red: HUVEC, blue: DAPI; Figure S5: Immunofluorescence images showing differences in CD31 expression in cross-sections of experimental groups. Green: CD31, red: HUVEC, blue: DAPI; Figure S6: Immunofluorescence images showing differences in Ang-1 expression in cross-sections of experimental groups at 1, 2, 4, and 8 weeks. Arrowheads indicate Ang-1 expression in images after staining. Dashed lines indicate edge of transplanted embossed sheets. Green: Ang-1, red: HUVEC, blue: DAPI. Scale bars are 200 μm; Figure S7: Immunofluorescence images showing differences in α-SMA expression in cross-sections of experimental groups. Dashed lines indicate edge of transplanted embossed sheets. Green: α-SMA, red: HUVEC, blue: DAPI; Figure S8: Images of embossed membrane with following conditions: (A) PCL membrane prepared by concentration (10 and 15%) in 1,1,1,3,3,3-hexafluoro-2-propanol (HFIP), (B) PCL membrane prepared by concentration (10 and 15%) in dimethylformamide (DMF)/tetrahydrofuran (THF), and (C) temperature of forming process with 12.5% polycaprolactone (PCL) membrane.
