# Supplementary material for: Embossed Membranes with Vascular Patterns Guide Vascularization in a 3D Tissue Model
Source: Polymers (Basel). 2019 May 2;11(5):792. doi: 10.3390/polym11050792 (PMC6572394; doi:10.3390/polym11050792)

*Supplement for*

# “Embossed Membranes with Vascular Patterns Guide Vascularization in a 3D Tissue Model”

Soyoung Hong, Eun Young Kang, Jaehee Byeon, Sung-ho Jung*and Changmo Hwang *

1. Figure S1: (A) Schematic of vacuum forming process for generation of embossed membrane. (B) Embossed membrane attached on polydimethylsiloxane mold. (C) Generated embossed polycaprolactone membranes. Supplemental data: Quantification of immunofluorescence staining using ImageJ software (% Area).

2. Supplemental data : Quantification of immunofluorescence staining using ImageJ software (% Area)

3. Figure S2: Preparation of cell sheet for cell culture. (A) Embossed membrane bonded onto frame having two notches, and (B) prepared in 6-well culture plate. (C) Cells were seeded onto both sides of membrane according to experimental procedures. (D) Stacking of embossed membrane was conducted using frame rig for easy assembly.

4. Figure S3: Hematoxylin and eosin-stained cross-section images of group 6x embossed after 2 weeks of implantation. (A) Region used for observation.

5. Figure S4: Immunofluorescence images showing differences in VE-cadherin expression in cross-sections of experimental groups at 1, 2, 4, and 8 weeks. Dashed lines indicate edge of transplanted embossed sheets. Green: VE-cadherin, red: HUVEC, blue: DAPI.

6. Figure S5: Immunofluorescence images showing differences in CD31 expression in cross-sections of experimental groups. Green: CD31, red: HUVEC, blue: DAPI.

7. Figure S6: Immunofluorescence images showing differences in Ang-1 expression in cross-sections of experimental groups at 1, 2, 4, and 8 weeks. Arrowheads indicate Ang-1 expression in images after staining. Dashed lines indicate edge of transplanted embossed sheets. Green: Ang-1, red: HUVEC, blue: DAPI. Scale bars are 200 μm.

8. Figure S7: Immunofluorescence images showing differences in $\alpha$-SMA expression in cross-sections of experimental groups. Dashed lines indicate edge of transplanted embossed sheets. Green: $\alpha$-SMA, red: HUVEC, blue: DAPI.

9. Figure S8: Images of embossed membrane with following conditions: (A) PCL membrane prepared by concentration (10 and 15%) in 1,1,1,3,3,3-hexafluoro-2-propanol (HFIP), (B) PCL membrane prepared by concentration (10 and 15%) in dimethylformamide (DMF)/tetrahydrofuran (THF), and (C) temperature of forming process with 12.5% polycaprolactone (PCL) membrane.

Figure S1: (A) Schematic of vacuum forming process for generation of embossed membrane. (B) Embossed membrane attached on polydimethylsiloxane mold. (C) Generated embossed polycaprolactone membranes. Supplemental data: Quantification of immunofluorescence staining using ImageJ software (% Area).

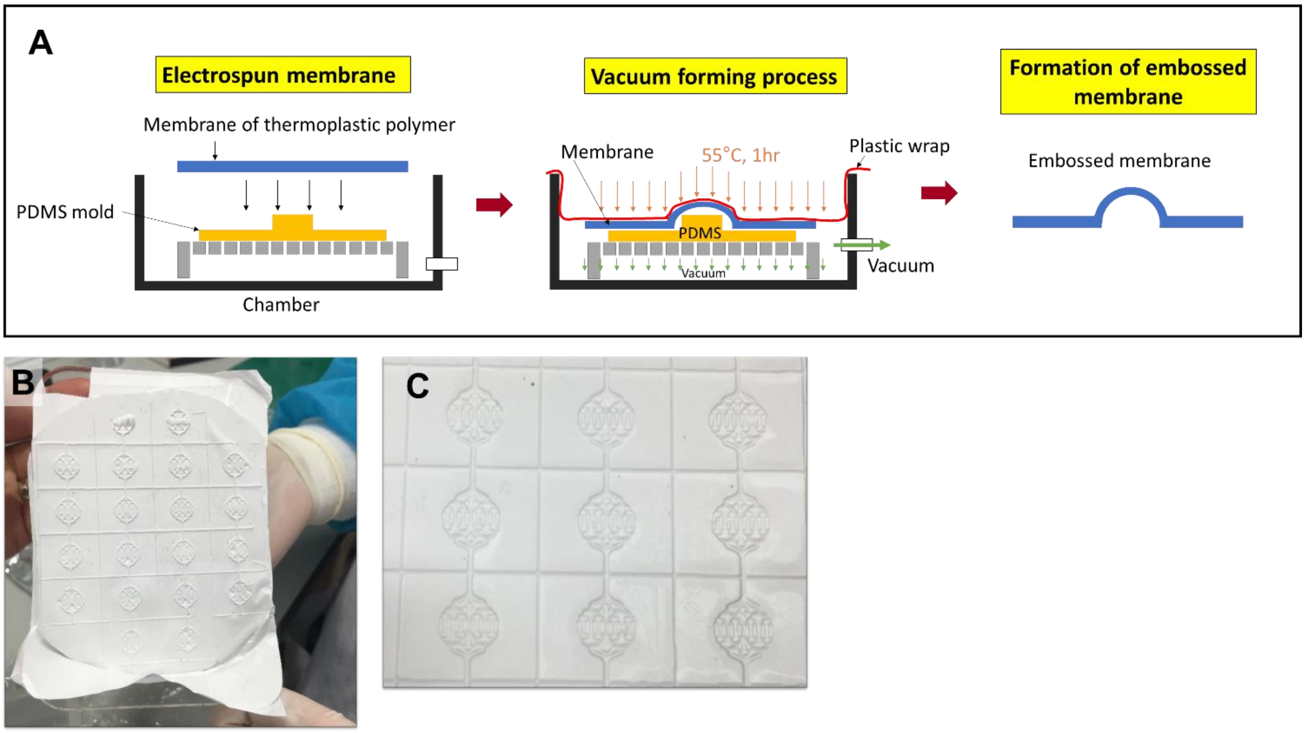

Supplemental data

***Quantification of immunofluorescence staining using ImageJ software (% Area)***

Digital images of stained cells were captured using confocal microscopy and processed in ImageJ in the following manner: (*1*) Using *Image* → *Color* → *Split Channels,* channels were separated for Green, red, or blue fluorescence channels. (*2*) Using *Image* → *Adjust* → *Threshold,* a constant threshold level was set across a set of conditions to ensure that enough signal was present to identify cells. (3) Using process → binary → make binary. (4) Using *Analyze* → *Tool* → *ROI Manager,* an ROI was selected as the area of interest using any of the drawing/selection tools. *(5)* Using *Analyze* → *analyze particles,* the area values were analyzed. The % area of experimental groups was expressed as a ratio of the area of red or green-fluorescent to the area mean of transplanted tissue sheet ROI.

Figure S2: Preparation of cell sheet for cell culture. (A) Embossed membrane bonded onto frame having two notches, and (B) prepared in 6-well culture plate. (C) Cells were seeded onto both sides of membrane according to experimental procedures. (D) Stacking of embossed membrane was conducted using frame rig for easy assembly.

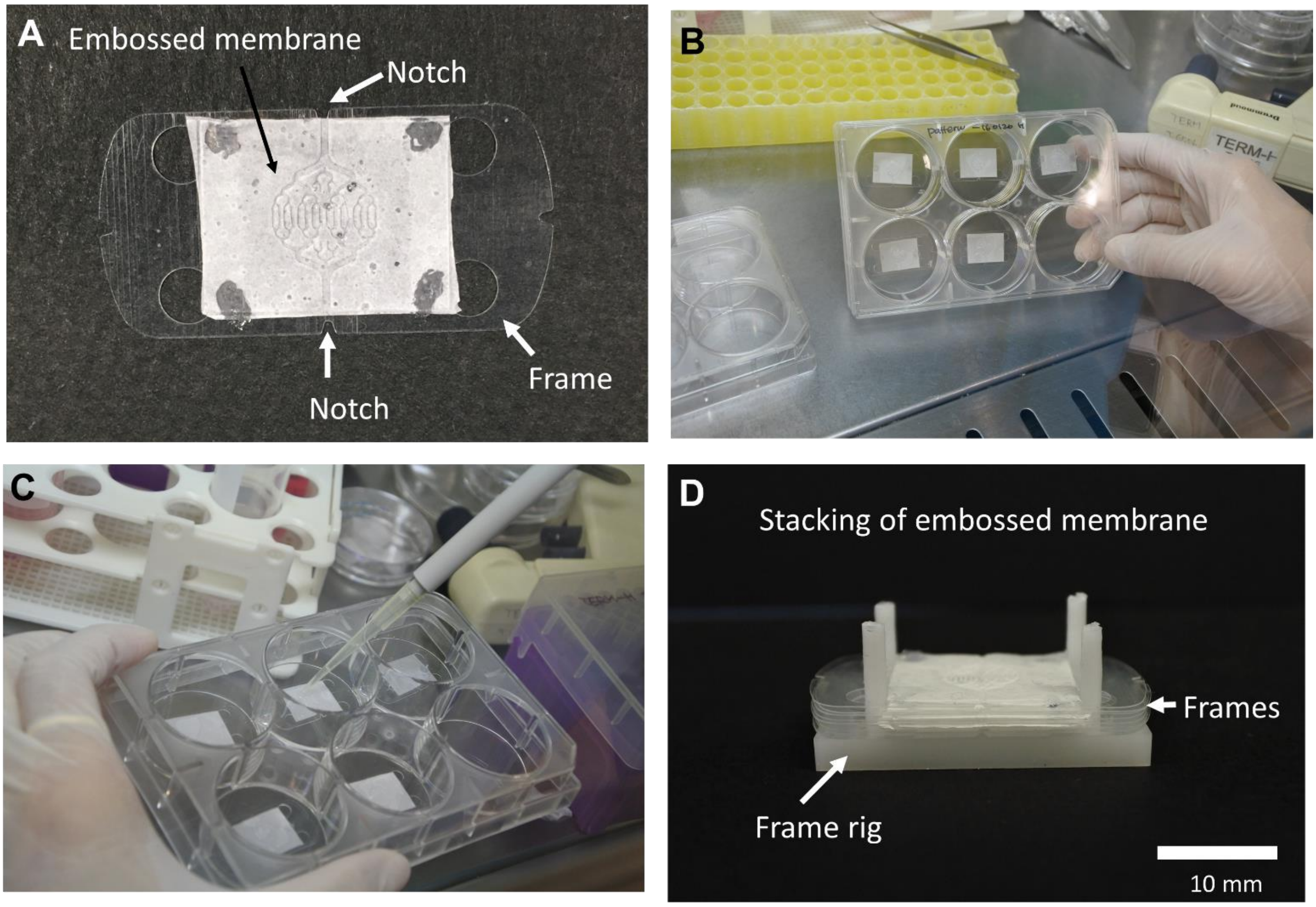

Figure S3: Hematoxylin and eosin-stained cross-section images of group 6x embossed after 2 weeks of implantation. (A) Region used for observation.

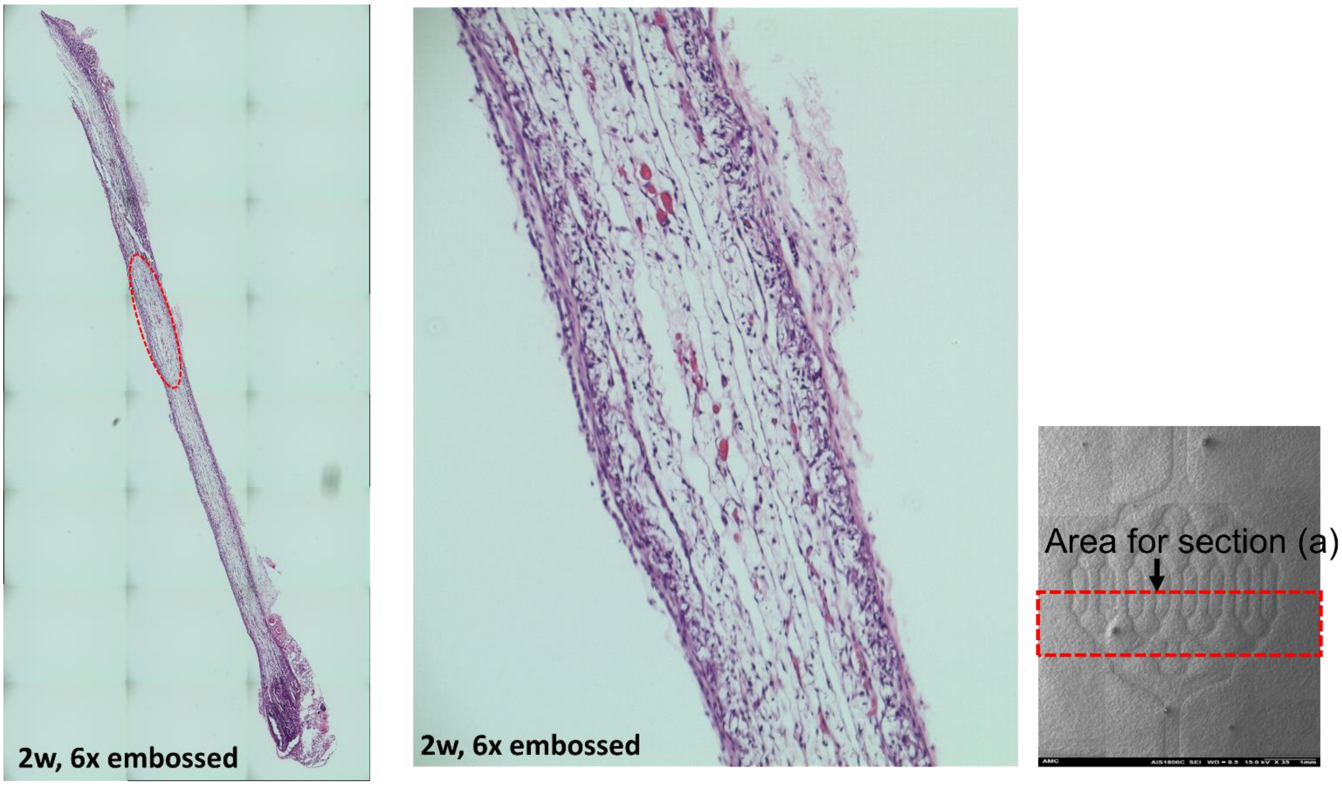

Figure S4: Immunofluorescence images showing differences in VE-cadherin expression in cross-sections of experimental groups at 1, 2, 4, and 8 weeks. Dashed lines indicate edge of transplanted embossed sheets. Green: VE-cadherin, red: HUVEC, blue: DAPI.

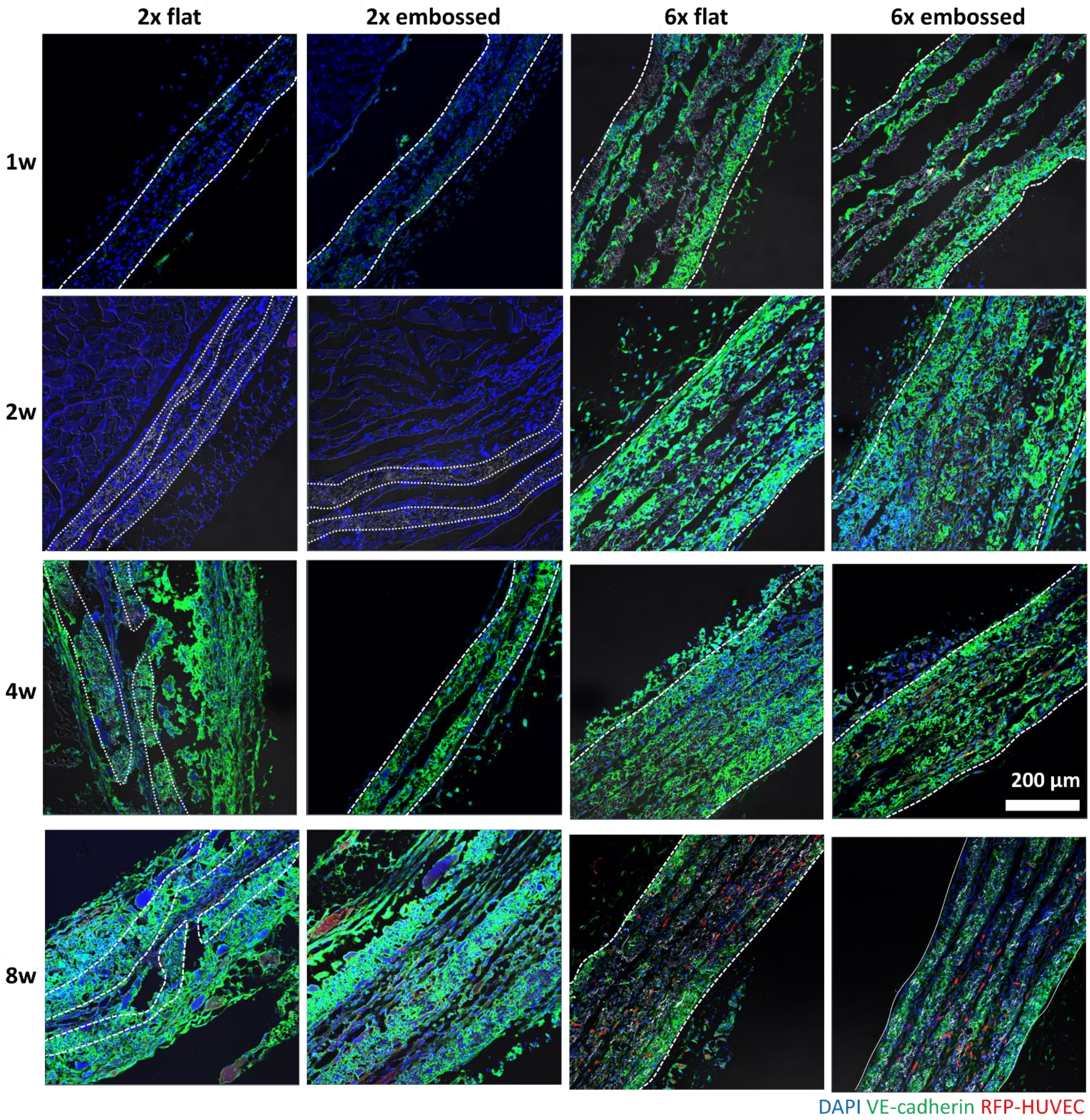

Figure S5: Immunofluorescence images showing differences in CD31 expression in cross-sections of experimental groups. Green: CD31, red: HUVEC, blue: DAPI.

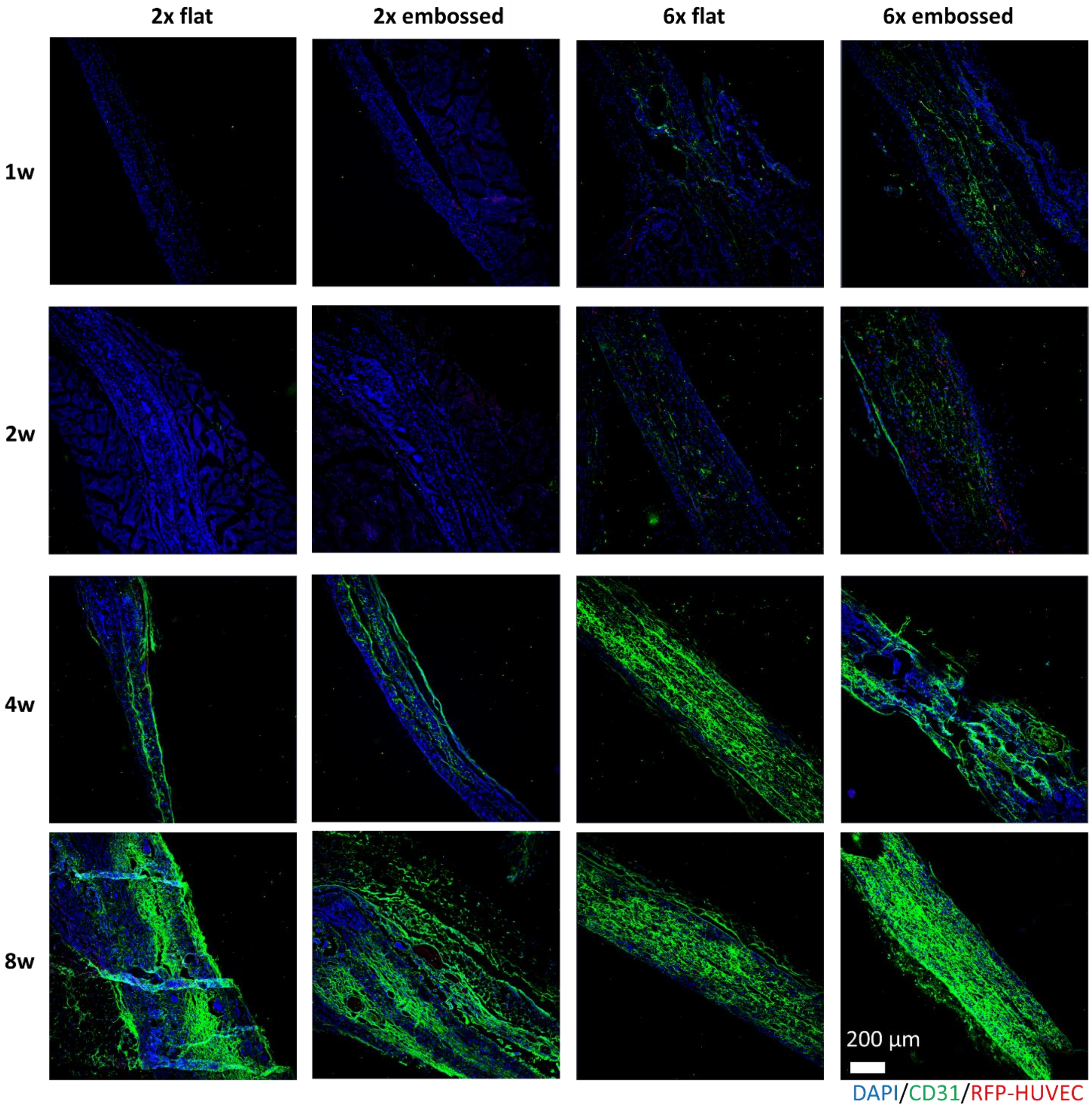

Figure S6: Immunofluorescence images showing differences in Ang-1 expression in cross-sections of experimental groups at 1, 2, 4, and 8 weeks. Arrowheads indicate Ang-1 expression in images after staining. Dashed lines indicate edge of transplanted embossed sheets. Green: Ang-1, red: HUVEC, blue: DAPI. Scale bars are 200 μm.

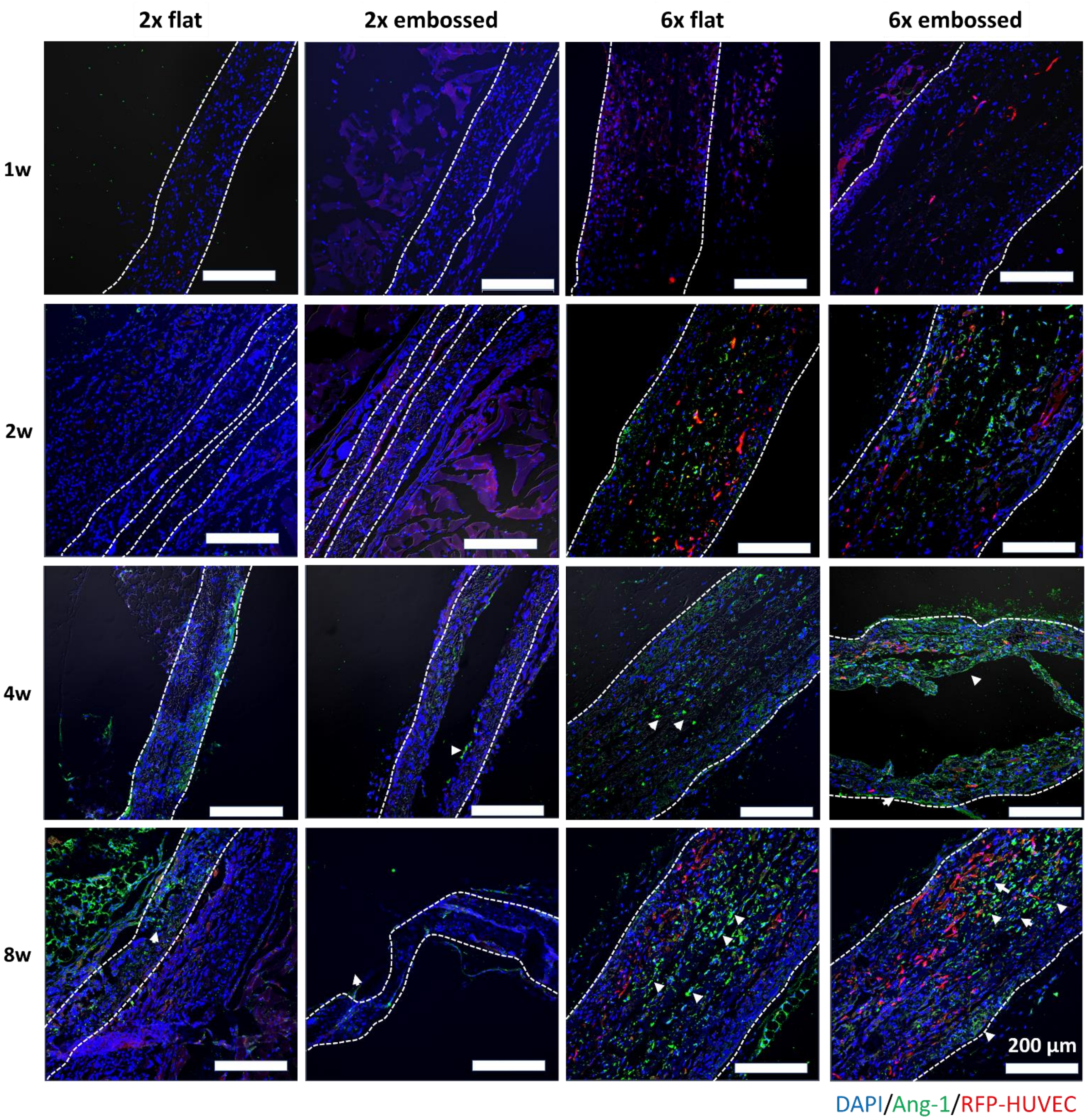

Figure S7: Immunofluorescence images showing differences in $\alpha$-SMA expression in cross-sections of experimental groups. Dashed lines indicate edge of transplanted embossed sheets. Green: $\alpha$-SMA, red: HUVEC, blue: DAPI.

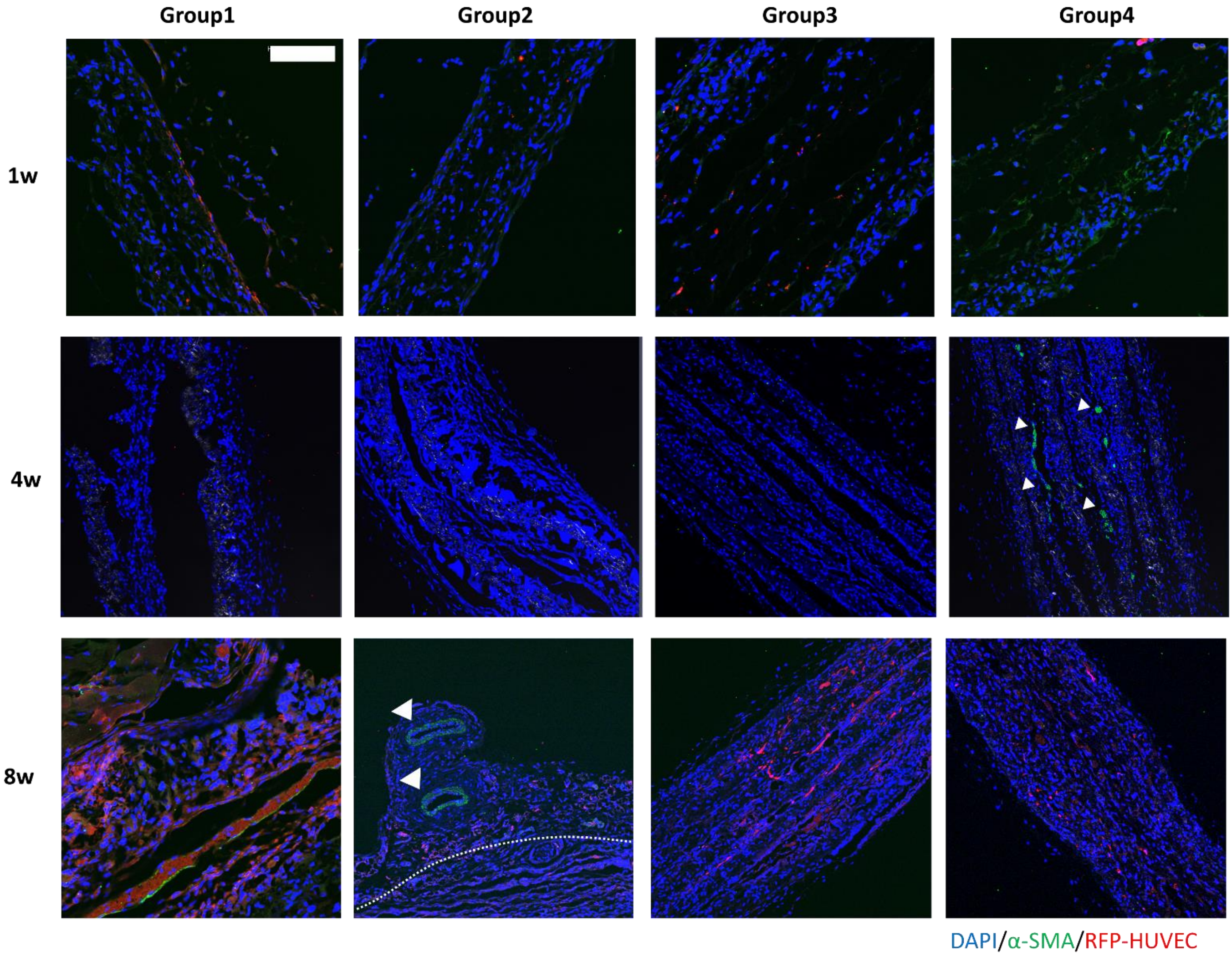

Figure S8: Images of embossed membrane with following conditions: (A) PCL membrane prepared by concentration (10 and 15%) in 1,1,1,3,3,3-hexafluoro-2-propanol (HFIP), (B) PCL membrane prepared by concentration (10 and 15%) in dimethylformamide (DMF)/tetrahydrofuran (THF), and (C) temperature of forming process with 12.5% polycaprolactone (PCL) membrane.

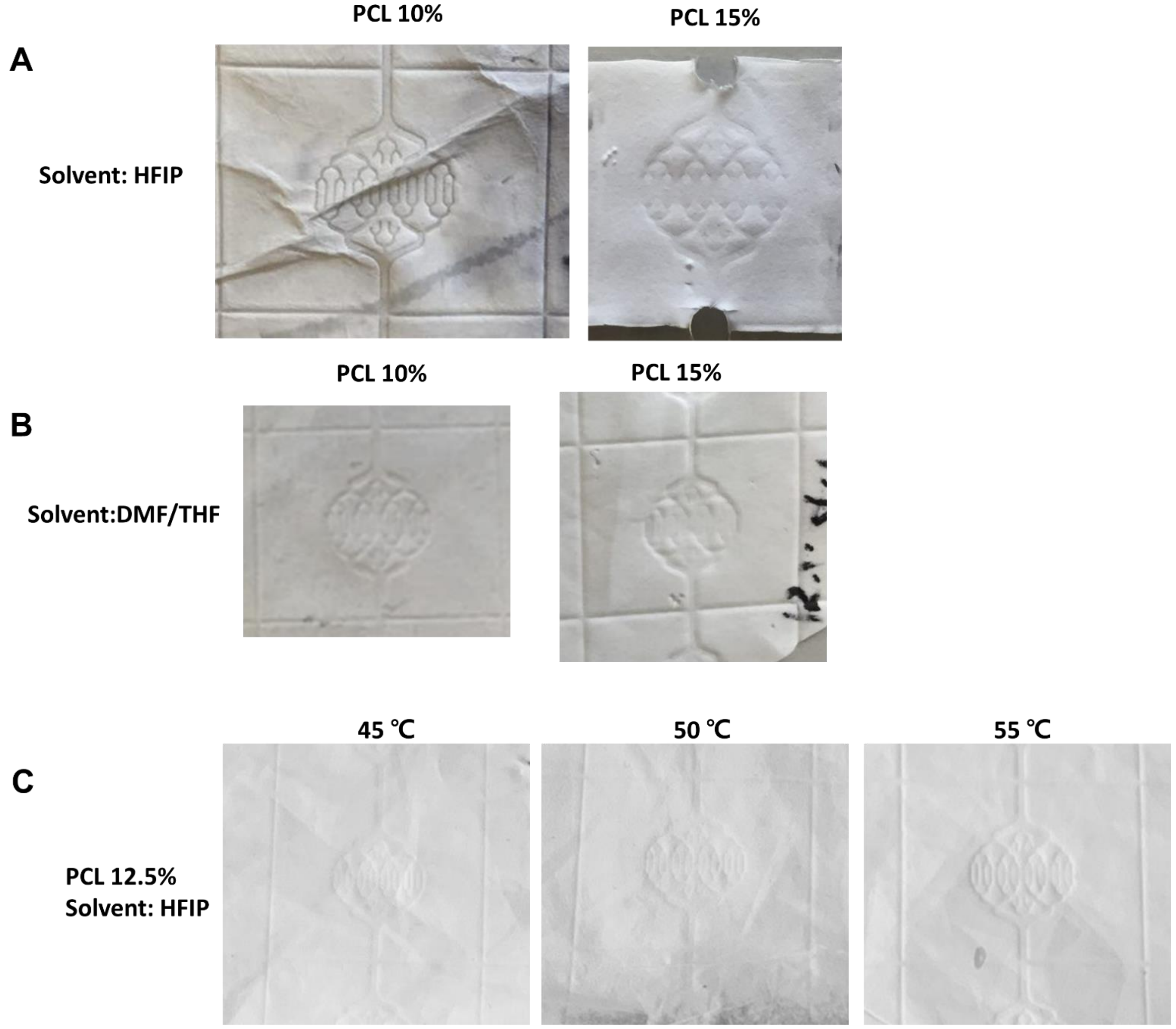

Supplement: Supplementary file 1 [file polymers-11-00792-s001.pdf]
